# Motivation to Participate in Precision Health Research and Acceptability of Texting as a Recruitment and Intervention Strategy Among Vietnamese Americans: Qualitative Study

**DOI:** 10.2196/23058

**Published:** 2021-03-11

**Authors:** Van Ta Park, Amber Kim, In Hyang Cho, Bora Nam, Khue Nguyen, Quyen Vuong, Vyjeyanthi S Periyakoil, Y Alicia Hong

**Affiliations:** 1 Department of Community Health Systems School of Nursing University of California, San Francisco San Francisco, CA United States; 2 School of Nursing San Jose State University San Jose, CA United States; 3 International Children Assistance Network Milpitas, CA United States; 4 Department of Medicine – Primary Care and Population Health School of Medicine Stanford University Stanford, CA United States; 5 College of Health and Human Services George Mason University Fairfax, VA United States

**Keywords:** Vietnamese Americans, texting, precision health, qualitative research, mobile phone

## Abstract

**Background:**

The largest effort undertaken in precision health research is the Precision Medicine Initiative (PMI), also known as the All of Us Research Program, which aims to include 1 million or more participants to be a part of a diverse database that can help revolutionize precision health research studies. Research participation from Asian Americans and Pacific Islanders in precision health research is, however, limited; this includes Vietnamese Americans, especially those with limited English proficiency. PMI engagement efforts with underserved communities, including members of minority populations or individuals who have experienced health disparities such as Vietnamese Americans with limited English proficiency, may help to enrich the diversity of the PMI.

**Objective:**

The aim of this study is to examine the attitudes towards and perceptions of precision health, motivations and barriers to participation in precision health research, and acceptability of SMS text messaging as a recruitment and intervention strategy among underserved Vietnamese Americans.

**Methods:**

A community sample of 37 Vietnamese Americans completed a survey and participated in one of 3 focus groups classified by age (18-30, 31-59, and ≥60 years) on topics related to precision health, participation in precision health research, texting or social media use experience, and insights on how to use text messages for recruitment and intervention. Participants were recruited via community organizations that serve Vietnamese Americans, flyers, word of mouth, and Vietnamese language radio announcements.

**Results:**

Most participants had little knowledge of precision health initially. After brief education, they had positive attitudes toward precision health, although the motivation to participate in precision health research varied by age and prior experience of research participation. The main motivators to participate included the desire for more knowledge and more representation of Vietnamese Americans in research. Participants were open to receiving text messages as part of their research participation and provided specific suggestions on the design and delivery of such messages (eg, simple, in both English and Vietnamese). Examples of barriers included misinterpretation of messages, cost (to send text messages), and preferences for different texting platforms across age groups.

**Conclusions:**

This study represents one of the first formative research studies to recruit underserved Vietnamese Americans to precision health research. It is critical to understand target communities’ motivations and barriers to participation in research. Delivering culturally appropriate text messages via age-appropriate texting and social media platforms may be an effective recruitment and intervention strategy. The next step is to develop and examine the feasibility of a culturally tailored precision health texting strategy for Vietnamese Americans.

## Introduction

### Background

Precision health is defined as *an innovative approach that considers individual differences in people’s genes, environments, and lifestyles* [1]. The largest effort in precision health research is the Precision Medicine Initiative (PMI or the *All of Us Research Program),* which aims to include 1 million or more participants to be a part of a diverse database that can help revolutionize precision health research [2,3]. The official launch of the *All of Us Research Program* was spring 2018 [4] and is anticipated to last for at least a decade. According to the All of Us Research Hub, which provides aggregate public data about the participants, the PMI currently includes 225,140 participants, as of February 11, 2020 [5].

However, when visiting the *All of Us* website (as of May 3, 2020) [6], it does not appear that non–English-speaking and non–Spanish-speaking racial and ethnic minorities, particularly Vietnamese Americans and persons with limited English proficiency (LEP), have been engaged. For example, there were no translated materials and videos in Vietnamese on the website. Moreover, when visiting the website [6], it appears that to enroll in the program, one needs to have an email address (and therefore access to the internet and a device such as a computer or smartphone).

It is important to ensure that underserved communities, defined by the Department of Health and Human Services as “communities that include members of minority populations or individuals who have experienced health disparities” [7], are engaged and educated on precision health. Asian Americans and Pacific Islanders (AAPI), for example, experience significant health and health care disparities [8], especially given that AAPI comprises 6% of the US population and is the fastest growing racial group in the United States [9]. They are heterogeneous in terms of languages and dialects (>100), cultural groups (>50), immigration patterns, religions, diets, and socioeconomic status [10]. Specifically, Vietnamese Americans have encountered premigration and migration traumas and other barriers (eg, low socioeconomic position, language difficulties) that place them at a higher risk for physical and mental illnesses compared with the general population [11-13]. As a relatively recent immigrant group, Vietnamese Americans are likely to be either first-generation or second-generation Americans, and there are about 2 million Vietnamese Americans in the United States [14]. They are the fourth largest foreign-born population from Asia (after China, India, and the Philippines) and have high rates of naturalization: about 76% of foreign-born Vietnamese Americans are naturalized US citizens.

Despite the growing AAPI population and the ongoing PMI, research participation from AAPI including Vietnamese Americans is limited. Of the 225,140 All of Us participants (database version February 11, 2020; 740/225,140, 0.33%) identified as Vietnamese [5]. Existing, limited research shows that AAPI are interested in being engaged but that various community concerns, such as a lack of culturally and linguistically appropriate information, need to be addressed to improve participation in clinical research and that engagement messages should include an *offer of hope* [15]. Moreover, a recent paper outlines the opportunities and challenges in precision medicine in improving cancer prevention and treatment for Asian Americans [16]. An integral point of their paper is to ensure that personalized medicine becomes a reality for all Americans [16].

### Objectives

Given the goals of the PMI to recruit 1 million persons into a precision health study of ≥10 years and that such persons should reflect the cultural diversity in the United States, it is important to explore strategies to meaningfully engage and educate racial and ethnic minorities about precision health and precision health research. Specific factors, such as English language proficiency, warrant special attention. Limited research exists on the feasibility or acceptability of using widely available mobile tools, such as texting for persons with LEP, including AAPI, for PMI-related purposes. Although traditional recruitment methods such as ethnic-specific radio/television advertisements have been successful in reaching out to Vietnamese Americans [17,18], this method has its limitations. The limitations include (1) not reaching every Vietnamese American as these audiences tend to be older in demographics, thereby limiting the ability to reach the younger generations; (2) high costs; and (3) the inability to reach the intended audience in *real time*, as it requires significant coordination with the radio/television producers/stations to get the messages out. It is critical to use a range of outreach methods, including those that are more cost-effective and wide reaching, to engage and educate Vietnamese Americans about precision health and precision health research. Accordingly, this study aims to examine the attitudes and perceptions of precision health, motivations and barriers to participate in precision health research, and acceptability of texting as a recruitment and intervention strategy among underserved English-speaking and Vietnamese-speaking Vietnamese Americans.

## Methods

### Recruitment

A community sample of 37 Vietnamese Americans comprising 3 age groups (18-30, 31-59, and ≥60 years) was recruited and participated in the study. During a 2-week period, 3 recruitment strategies were used: (1) announcements were made to the local community organizations that serve Vietnamese Americans, (2) flyers and word of mouth, and (3) Vietnamese language radio announcements. Given that one might have heard from multiple sources, 40 people reported that they heard about the study from friends or family (ie, word of mouth) or saw a flyer. A total of 30 people mentioned that they received announcements or heard directly through the primary Vietnamese community–based organization (ie, International Children Assistance Network [ICAN]), and 10 people learned about the study recruitment through a Vietnamese radio channel. The remaining channels were also reported: Facebook (n=3), email (n=3), and other (from a community outreach event; n=1).

A total of 73 persons expressed their interest to participate in the study. Out of the 73 people, 3 were not eligible for the following reasons: not available on the date of their age group (n=1), aged below 18 years (n=1), and called in to register after their age group had taken place (n=1). Of the 70 who were screened eligible to participate in the study, 33 did not participate because of the following reasons: withdrew owing to a last-minute schedule conflict (n=8), felt uncomfortable talking and answering questions in a focus group (3), had no transportation to the focus group (n=1), or wait-listed because the study reached its maximum number of preferred participants for the focus groups (n=21). The remaining 37 eligible participants were included in the study.

### Quantitative Data

After obtaining informed consent, participants were asked to complete a questionnaire pertaining to their sociodemographic background; questions were related to their knowledge, attitudes and behaviors in genetics and genetic testing, and texting attitudes for precision health. In total, 6 participants were able to fill their surveys directly on a web-based survey tool (ie, REDCap survey); however, the remaining 31 completed their surveys on hard copies because of a lack of internet access and/or a limited understanding on how to complete a web-based survey. Research staff later entered hard copy data onto REDCap.

### Sociodemographic and Texting Experience Questionnaire

We asked participants about their sociodemographic background, including their race, ethnicity, gender, year of birth, nativity, years lived in the United States, marital status, type of medical insurance, employment, education, household income and size, and English language proficiency.

Texting experience comprised questions, such as whether and how often they used a phone to send text messages, whether they used a phone to receive text messages, and which texting apps they used on their phones. We also asked participants if they had prior research participation experience.

### Genetics and Genetic Testing Survey

Participants were asked to indicate to what extent they agreed with the statements in the survey on a 5-point Likert scale from *strongly disagree* (*1*) to *strongly agree* (*5*). These items, which were drawn from previous precision health research with diverse racial/ethnic populations including Vietnamese Americans [18,19], included (1) genetics affect health; (2) the use of personal genetic information in health care is beneficial to patients; (3) I feel comfortable talking to my doctors about genetic testing; (4) the use of genetic tests could lead to discrimination; (5) if available, I would like to undergo genetic testing to find out if I am at risk of certain diseases (eg, cancer and diabetes); (6) if available, I would like to undergo genetic testing to find out if a certain drug (eg, high blood pressure medication) or treatment would work for my conditions; and (7) if available, I would like to participate in research about using genetics for disease prevention and treatment.

### Attitudes About Using Texting for the Precision Health Education Survey

Participants were asked to indicate to what extent they agreed with 3 statements to assess their attitudes about using texting for precision health education on a 5-point Likert scale from *strongly disagree* (*1*) to *strongly agree* (*5*). These items, which were developed for this study, included (1) I believe that text messages can help the community to understand how genetics affects health; (2) I believe that text messages can help the community to get genetic testing; and (3) I believe that text messages can help the community to understand what precision health is.

### Qualitative Data: Focus Groups

Three focus groups, which were separated by age groups, were conducted by the same bilingual research staff (QV and KN) in either English or Vietnamese. The focus group for participants aged 18 to 30 years (youngest age group) was conducted in the English language with 12 participants. The 2 focus groups for participants aged 31 to 59 years (middle-age group) and ≥60 years (oldest age group) were conducted in Vietnamese with 12 and 13 participants, respectively.

The facilitator (QV) from ICAN has significant prior experience facilitating focus groups. She began the focus group discussion by asking participants to share their knowledge regarding precision health and precision health research. After gauging their understanding, the facilitator explained what precision health and precision health research are. She then guided the focus group discussion. Each focus group lasted about 90 min, and the participants received a US $25 gift card to a local store for their time.

During the focus group, participants were asked to share (1) their knowledge of precision health and genetics; (2) knowledge, attitudes, and experiences with research; (3) perspectives and experiences with texting in general and with various texting apps; (4) insights on how to frame text messages on precision health for Vietnamese Americans; and (5) recommendations to best outreach to the Vietnamese community regarding precision health.

### Translation Process and Validity

The following materials were translated into Vietnamese: recruitment flyers, screening eligibility questions, informed consent, sociodemographic questionnaire, texting survey items, and focus group guide. The World Health Organization’s guidelines on translation and adaptation of instruments [20] were used to guide the translations of the study materials. By using this established translation method such as forward and backward translation methods, we were able to attain *conceptually equivalent* Vietnamese language versions of the English language materials, which were translated to be *cross-cultural and conceptual, rather than to be of linguistic/literal equivalence* [20]. Bilingual and bicultural Vietnamese research staff conducted the translations.

### Quantitative Data Analysis

Descriptive statistics were provided for the overall sample and focus group participation (age groups). Chi square and analysis of variance statistics were used to determine statistically significant differences among the groups. The data were analyzed using STATA 16 software [21].

### Qualitative Data Analysis

The focus group recordings were translated and transcribed verbatim into Microsoft Word documents. To increase the accuracy of the qualitative data, the research assistants conducted word-for-word translations of the focus group from Vietnamese to English. Content analysis was conducted on these transcripts by 2 raters. Using the questionnaire and common topics covered by the facilitator during each focus group session, a coding dictionary was created to be used as a guide. Subsequently, the 2 raters independently conducted thematic coding of the qualitative data using the thematic analysis approach by Luborsky [22]. After the initial content analysis, the raters discussed discrepancies in coding and agreed upon the emergent themes and subthemes (Textbox 1). The summary and reporting of the qualitative data were guided by examples from the literature on how to present qualitative findings [23] and examples from previous research that used qualitative methods to study vulnerable populations [24,25].

### Human Subjects Protection

This research was approved by the Stanford University Institutional Review Board (protocol no. 51409). Informed consent was obtained from all participants before study participation.

## Results

### Sample Characteristics

A total of 37 Vietnamese Americans participated in 3 focus groups by age groups: 18 to 30, 31 to 59, and ≥60 years (Table 1). Overall, slightly more females than males participated, but there was no significant difference by age group for gender. Overall, more than half (21/37, 57%) were married or living with a partner, with the remaining reporting being single (11/37, 30%) or separated/divorced/widowed (5/37,14%). There were significant differences by marital status for the age groups, with a high proportion (88%) of the youngest age group (18-30 years) reporting being single.

Approximately three-quarters (29/37, 78%) of the participants had acquired at least some college education. Regarding employment status, more than half (21/37, 57%) reported that they were employed either full time or part time, with the remaining stating that they were disabled/retired (10/37, 27%), a homemaker (3/37, 8%), unemployed (2/37, 5%), or a student (1/37, 3%). Household income was also reported, with more than half (22/37, 59%) of the participants making less than US $25,000 in 2018 for the entire household, and this stayed with the highest proportions (above 50%) among all 3 age groups.

There were significant differences by nativity, with most participants being born in Vietnam (34/37, 92%). Three US-born participants belonged to the youngest age group. About half (20/37, 54%) reported that they could speak at least some English and about half (17/37, 46%) could speak fluent or native English. The youngest age group tended to be more fluent in the English language and was the only group with participants who self-identified as native English speakers. The level of English fluency was affected by age groups. For instance, a high proportion (10/12, 83%) of the youngest age group stated being fluent in the English language, whereas only 33% (4/12) of the middle-aged participants and 25% (3/12) of the older age group stated this. On the contrary, when reporting English fluency as *some English*, the older age group (10/13, 77%) and middle-aged group (8/12, 67%) tended to have higher proportions.

Most participants (27/37, 73%) reported that they sent text messages regularly, with the highest proportion (12/12, 100%) from the youngest age group who reported that their phones can receive text messages (36/37, 97%). Phone texts (n=26) and Facebook (n=31) were the most common texting apps used among the participants, other apps included Viber (n=18), WhatsApp (n=5), WeChat (n=3), and others (n=8).

**Table 1:** Sample characteristics by age groups (N=37).

Characteristics		Total (N=37)	Age group (years)				*P* value	
			18-30 (n=12)	31-59 (n=12)	≥60 (n=13)			
**Age (years)**								<.001
	Mean (SD)	48.8 (19.3)	25.8 (3.0)	49.3 (10.1)	69.5 (4.4)			
	Median	52	27	53.5	68			
	Range	21-76	21-29	32-59	62-76			
**Gender, n (%)**								.62
	Female	22 (59.5)	6 (50)	7 (58.3)	9 (69.2)			
	Male	15 (40.5)	6 (50)	5 (41.7)	4 (30.8)			
**Current marital status, n (%)**								<.001
	Single	11 (29.7)	10 (83.3)	1 (8.3)	0 (0)			
	Married/living together	21 (56.8)	2 (16.7)	9 (75)	10 (76.9)			
	Separated/divorced/widowed	5 (13.5)	0 (0)	2 (16.7)	3 (23.1)			
**Education, n (%)**								.15
	Less than high school	2 (5.4)	0 (0)	2 (16.7)	0 (0)			
	High school	6 (16.2)	2 (16.7)	3 (25)	1 (7.7)			
	Some college	10 (27)	2 (16.7)	1 (8.3)	7 (53.8)			
	College or more	15 (40.5)	5 (41.7)	5 (41.7)	5 (38.5)			
	Graduate	4 (10.8)	3 (25)	1 (8.3)	0 (0)			
**Employment status, n (%)**								<.001
	Full time	15 (40.5)	9 (75)	6 (50)	0 (0)			
	Part time	6 (16.2)	1 (8.3)	3 (25)	2 (15.4)			
	Homemaker	3 (8.1)	0 (0)	2 (16.7)	1 (7.7)			
	Unemployed	2 (5.4)	1 (8.3)	1 (8.3)	0 (0)			
	Disabled/retired	10 (27)	0 (0)	0 (0)	10 (76.9)			
	Student	1 (2.7)	1 (8.3)	0 (0)	0 (0)			
**Household income (US $), n (%)**								.65
	<25,000	22 (59.5)	6 (50)	7 (58.3)	9 (69.2)			
	25,000-75,000	9 (24.3)	3 (25)	3 (25)	3 (23.1)			
	75,001-150,000	5 (13.5)	3 (25)	1 (8.3)	1 (7.7)			
	>150,000	1 (2.7)	0 (0)	1 (8.3)	0 (0)			
**Nativity, n (%)**								.03
	US-born	3 (8.1)	3 (25)	0 (0)	0 (0)			
	Foreign-born	34 (91.9)	9 (75)	12 (100)	13 (100)			
**English fluency, n (%)**								.006
	Native English	4 (10.8)	4 (33.3)	0 (0)	0 (0)			
	Fluent English	13 (35.1)	6 (50)	4 (33.3)	3 (23.1)			
	Some English	20 (54.1)	2 (16.7)	8 (66.7)	10 (76.9)			
**Send text messages, n (%)**								.12
	Regularly	27 (73)	12 (100)	8 (66.7)	7 (53.8)			
	Sometimes	8 (21.6)	0 (0)	3 (25)	5 (38.5)			
	Not often	2 (5.4)	0 (0)	1 (8.3)	1 (7.7)			
**Receive text messages, n (%)**								.39
	Yes	36 (97.3)	12 (100)	12 (100)	12 (92.3)			
	No	1 (2.7)	0 (0)	0 (0)	1 (7.7)			
**Types of text messages^a^, n (%)**								.09
	Phone texts	26 (70.3)	11 (91.7)	9 (75)	6 (46.2)			
	WeChat	3 (8.1)	2 (16.7)	1 (8.3)	0 (0)			
	WhatsApp	5 (13.5)	3 (25)	2 (16.6)	0 (0)			
	Facebook	31 (83.8)	11 (91.7)	12 (100)	8 (61.5)			
	Viber	18 (48.6)	4 (33.3)	11 (91.7)	3 (23.1)			
	Other^b^	10 (27)	5 (41.7)	2 (16.6)	3 (23.1)			

^a^Multiple-answer question.

^b^Other: Zalo, Snapchat, Skype, Line, Instagram, and Discord (n=10).

### Overall and by Genetic Testing and Texting

Table 2 shows the knowledge-attitude-behavior (KAB) scores within each group. The mean KAB score ranged from 1 (strongly disagree) to 5 (strongly agree). The overall mean KAB score was 3.95 (SD 0.36). The mean genetic testing score was 3.94 (SD 0.41), and the mean texting summary score was 3.96 (SD 0.63). There were no significant differences by age groups.

**Table 2:** Mean difference in summary scores by age groups: overall, genetics and genetic testing, and texting (N=37).

Category	Total (N=37)	Age group (years)				*P* value
		18-30 (n=12)	31-59 (n=12)	≥60 (n=13)		
Overall, mean (SD)	3.95 (0.36)	4.00 (0.98)	4.00 (0.10)	3.86 (0.11)	.55	
Genetics, mean (SD)	3.94 (0.41)	3.96 (0.13)	3.96 (0.09)	3.91 (0.13)	.35	
Texting, mean (SD)	3.96 (0.63)	4.08 (0.18)	4.08 (0.18)	3.74 (0.61)	.09	

 

### Qualitative Findings

The main themes from the qualitative data included the following: (1) perceptions of precision health, (2) motivation to participate in the research study, (3) advantages of texting, (4) barriers to texting, and (5) recommendations for texting strategies for precision health with Vietnamese Americans (Textbox 1).

**Overall qualitative themes and subthemes.**

Perceptions of precision health

- Genetic predisposition
- Lack of knowledge on precision health
- Usage as preventative measure
- Environmental factors

Motivation to participate in research study

- Representation of the Vietnamese American population
- Desire for knowledge
- Desire to contribute to research
- Previous experiences in participating in research studies
- Incentives
- Credibility of the research study or researcher

Advantages of texting

- Time efficiency and convenience
- Group messages
- Ease of sharing multimedia

Barriers to texting

- Misinterpretation
- Cost and/or need for Wi-Fi or mobile data
- Differing texting platforms among age groups
- Spam messages

Recommendations for texting strategies

- Short and concise messages
- Frequency—at most 1 to 2 messages per week
- Inclusion of pictures or links with brief descriptions
- Maintain respondents’ confidentiality
- Flexibility in language selection
- Simple language; use minimal medical, professional terminology (in English if necessary)
- Ability to forward or share information from texts
- Credible organizations and sources

#### Perceptions of Precision Health

Qualitative findings revealed that several participants expressed their perceptions of precision health and stated that precision health involves the study of genetic predisposition and use of genes to find specific treatments. The perceptions of precision health were similar in all 3 age groups, as participants discussed the association between family history, genes, and diseases.

Although some participants had previously heard of precision health, other participants reported a lack of knowledge in precision health and desired to learn about the meaning of precision health. Lack of knowledge on precision health was found in all 3 age groups; however, it was more prevalent in the youngest age group. Participants discussed that precision health can be used as a tool to prevent certain diseases. The discussions in all 3 age groups revealed participants’ understanding of precision health as a preventative measure to reduce the risk of diseases. In addition, some (n=5) reported that environmental factors, such as occupation and lifestyle, play important roles in determining a person’s health.

#### Motivation to Participate in Research

The participants reported that they were motivated to participate in research studies because they wanted more Vietnamese American representation in the field of research. One participant stated:

...but the most important thing is this research study is for Vietnamese Americans and Asian Americans. We (need to) participate to have a voice in the area of research so that researchers can learn what diseases are relevant to Vietnamese Americans and Asian Americans so they can tailor the treatments appropriately.

Participant 17, female, 52 years old

The participants believed that more representation of Vietnamese Americans was needed. The motivation to participate in research studies also reflected the participants’ desire for more knowledge in the research topics (n=13). The desire for knowledge was relevant in all 3 age groups. Previous participation in research studies also motivated several participants (n=4) to participate in this particular research study. Some participants (n=5) stated that incentives also influenced their motivation to participate in this study. Finally, a few (n=2) participants stated that the credibility of the research (ie, trusting the researcher, community-based organization, and/or university) may motivate them to participate in the research.

#### Advantages of Texting

The most common advantage noted across all 3 focus groups was the convenience and time efficiency of texting (*n*=12). One participant summarized the effectiveness of texting:

I would say we get an instant alert right away. If something happens, it’s just one text that we can get the information right away. I prefer texting over calling nowadays because I’m always busy with doing something, so I’d rather have someone text me and I’d get back to them right away.

Participant 10, male, 27 years old

Two participants noted their preferences for texting over sending emails:

I only check emails every 2-3 days. For texts, when we hear ding, we (can) open and read immediately.

Participant 37, female, 67 years old

I feel like when I get a text, it’s for me, so I prefer texts than emails.

Participant 31, female, 64 years old

Other common advantages included features of text messages, such as group messaging (n=2), confidentiality (n=1), and the ease of sharing multimedia via texting (*n*=3). Interestingly, a participant from the age group 18 to 30 years noted the sense of autonomy that texting provided:

Just going back to it fits your schedule. If you get a text and you want to reply to it, you can. If you don’t want to reply to it, you can put it off and lie, say you didn’t get it. Or just not respond.

Participant 8, male, 22 years old

From the older age group (≥60 years) in particular, a few participants (n=3) remarked on the more widespread access to cellphones than other devices such as computers.

#### Barriers to Texting

Several participants (n=5) reported the misinterpretation of text messages by the receiver as a barrier. Causes of misinterpretation included being unable to convey the tone of voice that would normally be conveyed if having a conversation in person. Text messages may also be disregarded as spam messages. Participants (n=3) from the older age group also brought up the varying use of tone symbols when typing in Vietnamese and abbreviated words as causes for misinterpretation. One participant provided an example stating:

Especially Vietnamese, people would abbreviate “mọi người” as “mn”, I don’t know if they mean “miền nam” (“South”) or something else.

Participant 37, female, 67 years old

In addition, participants (n=5) noted that the use of varying texting platforms could create difficulty in texting people, even among just the Vietnamese American population. One of the participants stated:

I would say it depends on the group of people you try to reach out to as I know my parents would use Viber, but they don’t text, for some reason. But it’s pretty much the same method of sending messages out. They prefer to use Viber because their friends are using Viber. For me I don’t really text with the (mobile) carrier, but I use Facebook messenger. So, it really depends on the group that you hang out with or that you work with. I’d prefer a different way of sending out messages.

Participant 10, male, 27 years old

When asked which texting services were used by the participants in each focus group, a list of apps was compiled, including Facebook Messenger, Viber, Zalo, Line, Google Voice, WhatsApp, Kakao Talk, and mobile carrier texts.

Concerns about cost and the need for Wi-Fi and/or mobile data were also suggested as barriers to texting (n=5). One participant from the youngest age group stated:

I’m actually annoyed by those internet-based apps because you always have to have Wi-Fi or your data on and if you don’t then there is a serious delay to the message. So, the contacts where we both have messenger, I don’t get their messages or otherwise unless the data is on or I have access to internet.

Participant 9, male, 28 years old

Other barriers put forth by the participants were having to switch between English and Vietnamese keyboards (n=1), autocorrect (n=2), and the lack of knowledge regarding how to set up different texting apps (n=1).

#### Recommendations for Texting Strategies

Several participants (n=8) from all 3 age groups discussed the need for a concise, simple language when texting health information. They recommended the use of minimal medical or professional terminology for one of the texting strategies. One participant stated:

I’d say use simple language because he mentioned earlier that some medical terminology might be too complicated for someone to understand what it means. So, try to use a daily language, it’d be helpful.

Participant 10, male, 27 years old

Don’t use too many medical terms.

Participant 21, male, 32 years old

In addition, participants (n=8) stated that the frequency of text reminders should not exceed more than 1 or 2 text messages in 1 week. If the text messages were sent frequently, the participants were likely to consider the text messages irrelevant. One participant from the youngest age group stated:

I think it's also very important who the message is coming from...But if it was another entity, or I had no idea who they were, I would have felt annoyed at the frequency because I got 3 messages as a reminder for this and an email.

Participant 7, female, 28 years old

Participants (n=2) from the older age group emphasized the inclusion of pictures, links, and brief descriptions of relevant health information when sending text messages.

Several participants (n=6) expressed concerns regarding confidentiality and security when receiving text messages. They stated that it is necessary to maintain confidentiality when sending text messages involving personal information. This concern was common in all 3 age groups, as 1 participant from the youngest age group reported:

Like if you send the important information, health conditions of one person through texting that is kinda not formal. I don’t feel it’s safe to send that information through texts.

Participant 6, female, 26 years old

Another recommendation is language selection. Several participants (n=6) suggested the need to offer language preferences because of the diversity in experience and background. However, when using Vietnamese, it needed to be written correctly in grammar and form to avoid misunderstanding. The facilitator confirmed this understanding when she stated, *“*The content should be in Vietnamese, but the terminology would be also included in English.” One participant replied, “That’s correct” (participant 31, female, 65 years old). Another participant added, “Vietnamese with tones” (participant 30, male, 73 years old).

In addition, when the focus group facilitator asked the participants (in the 60 years old and older focus group) on whether medical terminology should be translated, they responded, “No need to translate.” In other words, it is best to keep its given name and translate the concept instead. This was further supported by participants in the other focus groups (18-30 and 31-59 years old):

If the translation seems odd or tricky, then include the English term.

Participant 15, female, 58 years old

There are a lot of direct translations of English like legal or judicial or medical terms sometimes they are very off.

Participant 9, male, 28 years old

Participants (n=2) from the middle-aged group discussed their ability to forward or share relevant information from texts as one of the texting strategies. One participant stated:

When I see health information on Facebook, I would select the ones that would be of great benefit to me and then I would share them and keep them for myself...If I see them as helpful, such as exercise for elders or new treatments. Of course I always select them carefully if I want to keep for myself and forward to friends.

Participant 18, female, 59 years old

## Discussion

### Summary of Findings and Comparison With Previous Research

This pilot study is the first of its kind to examine the attitudes and perceptions of precision health and precision health research while also exploring a novel method of recruitment (ie, texting) among an underserved racial population in the United States, especially Vietnamese Americans. Specifically, the study explored motivations and barriers to research participation and cultural acceptability of texting as a recruitment and intervention strategy for precision health research.

The overall findings show that although many of the participants reported being familiar with genetics and genetics testing, some reported lack of knowledge in precision health or precision medicine and desired to learn more about these topics. Once the participants were more informed about precision health (through the facilitator’s introduction of the topics), they became more receptive and expressed positive attitudes toward participating in precision health research and provided input on how texting could be used to recruit and engage Vietnamese Americans in research. Although there is limited prior research on this topic, there was 1 qualitative study (where the lead author of this paper was a collaborator) that had also employed focus groups among American Indian, African American, Latino, Chinese, and Vietnamese groups and found similar findings (ie, limited prior knowledge about precision health) [19]. Moreover, the same study reported that all Latino, Chinese, Vietnamese, and some African American participants had positive attitudes toward precision health [19].

Qualitative themes indicated that although it slightly varied among the 3 age groups, most participants were motivated to participate in research because of the lack of the Vietnamese American representation in the area of research, and they expressed how they would want to contribute to this effort (to increase research participation). In addition, many participants reported a desire to gain more knowledge in their research.

The study findings support the notion that to engage Vietnamese Americans in research (eg, precision health research), the community’s needs must be met. For example, Vietnamese Americans need to understand the specific benefits of research participation personally and for their communities [26]. Moreover, research needs to be culturally tailored for Vietnamese Americans in language and cultural beliefs/norms [26,27].

Participants responded positively to using texting as a recruitment strategy for precision health research. They indicated that they prefer texting over other communication methods (eg, emails) because it is convenient, time efficient, low cost, and widely used. About three-quarters of the study participants reported that they sent text messages regularly, and most of them received text messages. The 2 most commonly reported texting methods were Facebook (n=31) and phone texts (n=26). Although previous research has not focused on texting as a strategy for precision health research, it has highlighted the acceptability of text messages for delivering health messages to different ethnic minority populations [28-30]. Furthermore, a recent systematic review found that few studies have examined the use of digital tools (eg, texting) for the recruitment and retention of racial/ethnic minority participants in intervention research and that randomized controlled trials are limited. Thus, the next phase of our research may play an important role in contributing to our understanding of the potential utility of texting as a precision health research recruitment tool for racial/ethnic minorities such as Vietnamese Americans.

The participants offered recommendations to overcome potential barriers to implement texting as a recruitment strategy for precision health research. For example, a common barrier that participants brought up was the misinterpretation of the meanings or intentions of the text messages, including a lack of tone of voice and the use of abbreviations, which are important in the Vietnamese language [31]. Moreover, it is important to select words that are easy to understand, and if using Vietnamese, words need to be culturally appropriate and have tone symbols, which is particularly relevant when providing appropriate services to people who speak Vietnamese[31]. These recommendations are consistent with previous research on other racial/ethnic populations. For example, the use of abbreviations, particularly abbreviated slang, would be difficult to understand among older Samoans [30].

### Strengths and Limitations

A strength of our study is that our focus groups were separated by age groups (18-30, 31-59, and 60 years and older) and language proficiency (English; Vietnamese). This enabled us to ensure diverse perspectives by age (young adults, middle-age adults, and older adults) and Vietnamese proficiency with LEP. Future research could involve focus groups being conducted with the same gender (ie, all males in one focus group and females in a different focus group) and/or semistructured interviews to potentially elicit shared perspectives by gender and/or permit the participants to provide detailed perspectives.

This study had several limitations. First, we conducted a qualitative study on a small sample of Vietnamese Americans. The data presented might not be generalizable to other Vietnamese Americans in the United States. Second, the discussion on using mobile tools for recruitment and research was focused more on texting and social media platforms, although other strategies might also be viable. Third, the feasibility and acceptability of using texting as a recruitment and intervention strategy were based on self-report attitudes in focus groups, which merits field testing to confirm the feasibility of conducting precision health research in the underserved Vietnamese American community.

### Conclusions

To improve the research participation among racial/ethnic minority populations and have equitable representation that mirrors the diversity of the United States in precision health research, there needs to be more thoughtful and committed efforts to engage them. These efforts include having culturally and linguistically tailored materials, age-appropriate texting strategies, and meaningful benefits to the Vietnamese American community. Furthermore, it is important to explore alternate ways of engaging with potential participants who do not have access to digital technology, such as email. Precision health research, and all research more broadly, which requires an email address for participation, would exclude many potential participants in marginalized populations. Our study provides support for the use of texting as an example of an alternate strategy to engage such participants.

As a next step of this formative research, we will be developing and piloting a culturally tailored texting strategy as an educational and engagement tool for Vietnamese Americans about precision health. In the next study phase, we will examine the acceptability of such a texting strategy and develop a library of culturally appropriate precision health messages that may be implemented on a large scale. We will partner with a large Vietnamese community–based organization for this important next phase of community-based participatory research.

## Abbreviations

AAPI: Asian Americans and Pacific Islanders
ICAN: International Children Assistance Network
KAB: knowledge-attitude-behavior
LEP: limited English proficiency
NIH: National Institutes of Health
PMI: Precision Medicine Initiative
